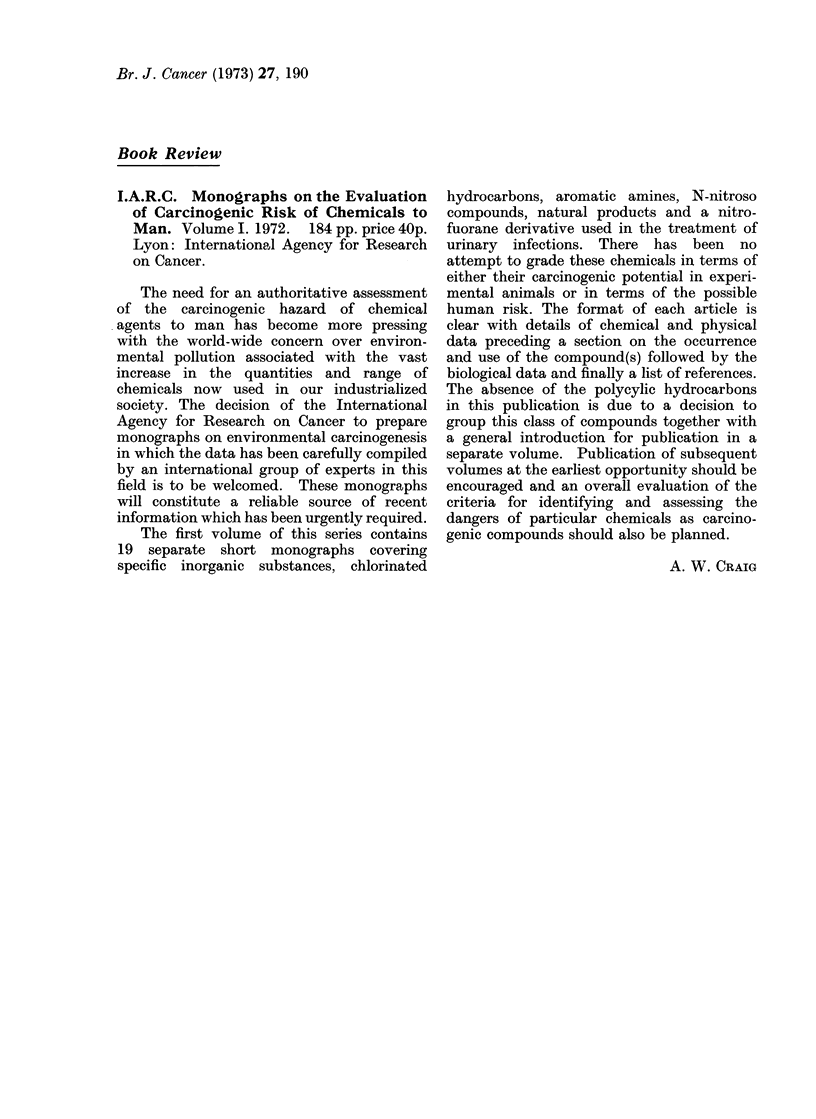# I.A.R.C. Monographs on the Evaluation of Carcinogenic Risk of Chemicals to Man

**Published:** 1973-02

**Authors:** A. W. Craig


*Br. J. Cancer* (1973) **27**, 190

### *Book Review*

**I.A.R.C. Monographs on the Evaluation of Carcinogenic Risk of Chemicals to Man.** Volume I. 1972. 184 pp. price 40p. Lyon: International Agency for Research on Cancer.

The need for an authoritative assessment of the carcinogenic hazard of chemical agents to man has become more pressing with the world-wide concern over environmental pollution associated with the vast increase in the quantities and range of chemicals now used in our industrialized society. The decision of the International Agency for Research on Cancer to prepare monographs on environmental carcinogenesis in which the data has been carefully compiled by an international group of experts in this field is to be welcomed. These monographs will constitute a reliable source of recent information which has been urgently required.

The first volume of this series contains 19 separate short monographs covering specific inorganic substances, chlorinated hydrocarbons, aromatic amines, N-nitroso compounds, natural products and a nitrofuorane derivative used in the treatment of urinary infections. There has been no attempt to grade these chemicals in terms of either their carcinogenic potential in experimental animals or in terms of the possible human risk. The format of each article is clear with details of chemical and physical data preceding a section on the occurrence and use of the compound(s) followed by the biological data and finally a list of references. The absence of the polycylic hydrocarbons in this publication is due to a decision to group this class of compounds together with a general introduction for publication in a separate volume. Publication of subsequent volumes at the earliest opportunity should be encouraged and an overall evaluation of the criteria for identifying and assessing the dangers of particular chemicals as carcinogenic compounds should also be planned.

A. W. Craig